# Atmospheric Warming Drives Growth in Arctic Sea Ice: A Key Role for Snow

**DOI:** 10.1029/2020GL090236

**Published:** 2020-10-24

**Authors:** A. Bigdeli, A. T. Nguyen, H. R. Pillar, V. Ocaña, P. Heimbach

**Affiliations:** ^1^ Oden Institute for Computational Engineering and Sciences University of Texas at Austin Austin TX USA; ^2^ Jackson School of Geosciences University of Texas at Austin Austin TX USA; ^3^ Institute for Geophysics University of Texas at Austin Austin TX USA

**Keywords:** Arctic sea‐ice sensitivity to atmospheric warming, numerical adjoint, sea‐ice growth, conductivity feedback

## Abstract

A number of feedbacks regulate the response of Arctic sea ice to local atmospheric warming. Using a realistic coupled ocean‐sea ice model and its adjoint, we isolate a mechanism by which significant ice growth at the end of the melt season may occur as a lagged response to Arctic atmospheric warming. A series of perturbation simulations informed by adjoint model‐derived sensitivity patterns reveal the enhanced ice growth to be accompanied by a reduction of snow thickness on the ice pack. Detailed analysis of ocean‐ice‐snow heat budgets confirms the essential role of the reduced snow thickness for persistence and delayed overshoot of ice growth. The underlying mechanism is a snow‐melt‐conductivity feedback, wherein atmosphere‐driven snow melt leads to a larger conductive ocean heat loss through the overlying ice layer. Our results highlight the need for accurate observations of snow thickness to constrain climate models and to initialize sea ice forecasts.

## Key Points

- Arctic sea ice sensitivity to atmospheric warming is computed using a numerical adjoint, revealing warming can lead to lagged sea ice growth
- Snow melt—in response to early warming—is the key preconditioner, enabling large conductive ocean heat loss to support lagged ice growth

## 1 Introduction

Recent observations reveal that sea ice volume in the Arctic is declining, and its distribution is shifting towards thinner and younger ice (Kwok & Rothrock, 2009; Laxon et al., 2013; Lindsay & Schweiger, 2015; Schweiger et al., 2019). These changes are thought to have occurred mainly in response to trends in local near‐surface air temperature (Olonscheck et al., 2019). It has been hypothesized that various ice‐snow‐ocean feedbacks may have amplified these changes (Rampal et al., 2011; Wang & Overland, 2009) or exerted a stabilizing influence (Tietsche et al., 2011). The proposed thermodynamic feedbacks can be divided into two categories, both of which are well documented in the literature (Massonnet et al., 2018; Stroeve et al., 2012). The positive, that is, destabilizing ice‐albedo feedback is a consequence of the lower albedo of open water compared to ice or snow. As the ratio of ice‐covered area to open water decreases, the radiative flux absorbed by the system increases (Timmermans et al., 2018). The negative, that is, stabilizing ice‐conductive feedback is a consequence of thinner ice (and snow) becoming more thermally conductive. When exposed to winter Arctic subfreezing air temperatures, enhanced conductivity enables faster sea ice growth (Bitz & Roe, 2004).

The relative importance of the ice‐albedo and ice‐conductivity feedbacks is difficult to determine from observations (Goosse et al., 2018) and remains an area of active research (Petty et al., 2018). Investigating observational records, Notz and Marotzke (2012) suggest that the strength of the stabilizing feedback currently exceeds that of the destabilizing feedback, in agreement with other studies (Eisenman & Wettlaufer, 2009; Tietsche et al., 2011). It implies that there may not be an irreversible loss of Arctic summer sea‐ice over the coming century, despite its recently observed rapid retreat.

General Circulation Models (GCMs) of various complexities have been used to examine several mechanisms underlying the observed trends in sea‐ice extent and to estimate the amplitude of existing feedbacks (e.g., Blanchard‐Wrigglesworth & Bitz, 2014; Li et al., 2013; Stroeve et al., 2018). Although it has been shown that the strengths of both ice‐conductivity and ice‐albedo feedbacks are largely comparable across these models (Massonnet et al., 2018, compared 44 GCMs), the assessment of their magnitude is based on correlations between metrics of the atmospheric forcing and sea‐ice state (Hegyi & Taylor, 2016, 2017; Petty et al., 2018; Stroeve et al., 2018), complicating the unambiguous identification of a causal relation between forcing and response.

In this study we use a regional, coupled ocean‐sea ice configuration of the Massachusetts Institute of Technology general circulation model (MITgcm, Marshall et al., 1997) and its adjoint (Heimbach et al., 2005) to establish a causal relationship between the total ice volume in the Arctic Ocean basin and local, near‐surface air temperature. The adjoint model provides the linear sensitivity of a scalar‐valued differentiable quantity of interest to all control variables, throughout the entire nonlinear model trajectory. These sensitivity fields evolve through space and time, thus revealing the mechanisms through which variability in the quantity of interest arises. Alternatively, in less technical terms, the adjoint can be loosely defined as a model that outputs the rate of change of a single model output variable in response to changes in each model input and/or parameter, throughout the simulation.

The MITgcm adjoint has been widely used for sensitivity studies to elucidate the timescales and pathways of variability in various circulation metrics, including Atlantic overturning circulation (Heimbach et al., 2011; Pillar et al., 2016, 2018; Smith & Heimbach, 2018), regional heat content (Jones et al., 2018), and integrated sea surface height (Verdy et al., 2013). Adjoint‐based investigation of sensitivities of the sea‐ice state has been less common. Relevant studies to date have examined mechanisms underlying variability in ice export through the Canadian Arctic Archipelago (Heimbach et al., 2010), regional Arctic sea ice extent (Kauker et al., 2009), and low frequency trends in Arctic ice volume (Koldunov et al., 2013). The adjoint‐enabled sea ice component of the MITgcm has been developed initially in order to enable coupled ocean‐sea ice state estimation (Fenty & Heimbach, 2013a, 2013b; Fenty et al., 2017; Koldunov et al., 2017).

Here, we integrate the sea‐ice adjoint model component into the Arctic and Subpolar gyre sTate Estimate (ASTE, Nguyen et al., 2017) to explore the mechanisms underlying the sea ice response to surface atmospheric temperature variations (section 2). In particular, we focus on how increases in September Arctic ice volume arise as a lagged response (over a 1 year period) to regional (Arctic) atmospheric warming through analysis of spatial patterns of ice volume sensitivity to surface atmospheric temperature perturbations (section 3). We present results from an ensemble of forward perturbation simulations, designed to support and elucidate the dominant feedback mechanisms underlying the observed sensitivity distributions. We discuss our results in the context of previous work and address the implications of our key findings for climate predictions, providing some directions for future investigation (section 4).

## 2 Methods

### 2.1 Ocean‐Sea Ice State Estimation Framework

For this study, we use the MITgcm in a regional coupled ocean‐sea ice configuration, very similar to that of the Arctic and Subpolar gyre sTate Estimate, release 1 (ASTE‐r1, Nguyen et al., 2017). ASTE has been developed within the state estimation framework of the “Estimating the Circulation and Climate of the Ocean” (ECCO) consortium (Forget et al., 2015a; Stammer et al., 2002; Wunsch & Heimbach, 2007). It provides an estimate of the time‐evolving, coupled ocean‐sea ice state that is kinematically and dynamically consistent with the conservation laws underlying the MITgcm formulation and (to within prescribed uncertainty) with O(10^6^) satellite and in situ observations from 2002 to 2017.

Our model configuration differs from ASTE‐r1 only in the coarser horizontal resolution (∼35 km in the Arctic vs. ∼14 km in ASTE‐r1) to make our computationally intensive adjoint studies tractable. The model is initialized from the ASTE‐r1 solution for year 2002, subsampled to match the coarser horizontal resolution. The atmospheric state is based on the JRA‐55 reanalysis (Kobayashi et al., 2015). Open‐ocean air‐sea fluxes are computed using the Large and Yeager (2009) bulk formulae.

The sea ice model consists of separate dynamic and thermodynamic components coupled to the ocean model. The dynamic component is based on the standard nonlinear viscous‐plastic rheology proposed by (Hibler, 1979), with improvements described by Losch et al. (2010). The thermodynamic component is based on the zero‐heat‐capacity formulation of Semtner (1976). In this formulation, an equilibrium system is assumed with a closed surface heat budget. Sea ice growth occurs via basal freezing. During freezing, heat is conducted upward through the system assuming a linear temperature profile and constant conductivity. Snow accumulation occurs via precipitation of rain on the subfreezing sea ice surface. If the weight of accumulated snow on the ice exceeds the sea ice buoyancy, the snow fraction depressed below the water line is adiabatically converted to sea ice. The model does not distinguish between different ice types (e.g., first year versus multiyear). While this is thought to be acceptable for simulating feedback mechanisms (Blanchard‐Wrigglesworth & Bitz, 2014; Massonnet et al., 2018), it has been shown that this plays a role in predicting thick to thin ice transitions and melting (Guemas et al., 2016).

### 2.2 Deriving Adjoint Sensitivities

The adjoint model provides an efficient means for computing the sensitivity of a scalar‐valued, differentiable model metric (the quantity of interest) to independent variables, that is, inputs to the model, throughout the model integration. Our quantity of interest is the monthly mean sea ice volume (*V*_*SI*_) integrated over the entire Arctic Ocean for the month of September, when the seasonal minimum occurs. Our control variable is the atmospheric near‐surface (*z* = 2 m) air temperature (*T*_*air*_). Monthly mean linear sensitivities, *∂V*_*SI*_/*∂T*_*air*_, as a function of space and (lagged) time are calculated for the entire model trajectory (2002–2015). Since our results are insensitive to the year in which the cost function is defined (not shown), we focus on September 2006 as our quantity of interest, *V*_*SI*_. Our choice of 2006 is arbitrary and has no qualitative impact on the sensitivity pathways or feedback mechanism presented below.

**Figure 1:**
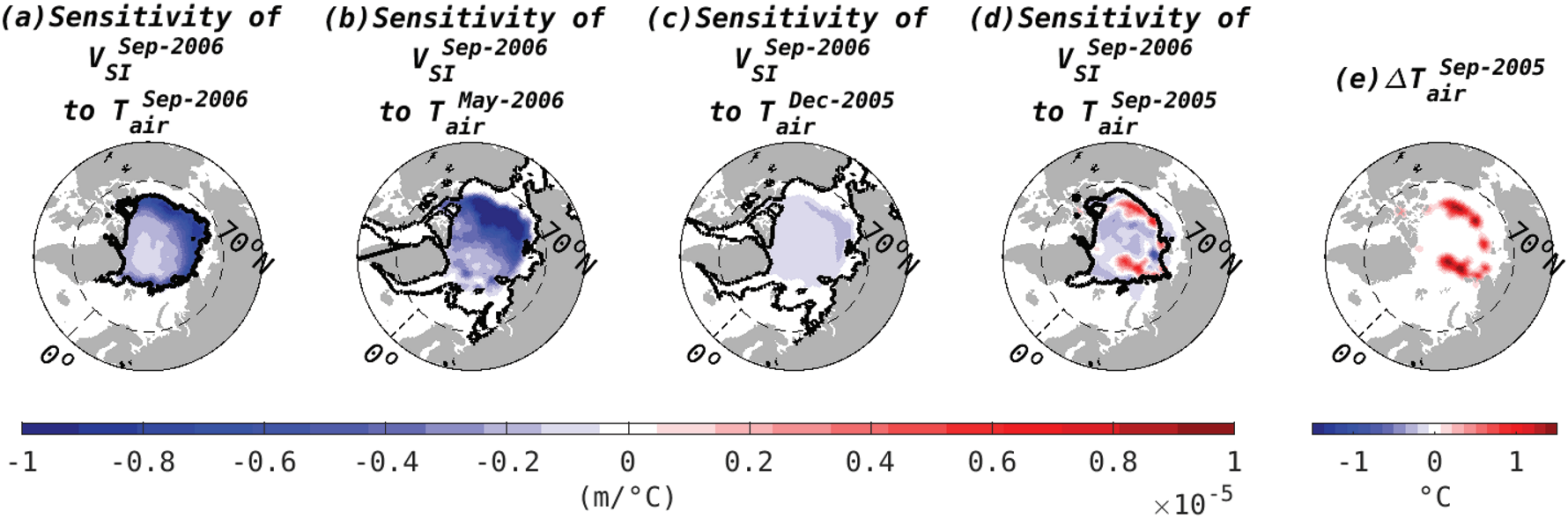
Linear sensitivity, *∂V*_*SI*_/*∂T*_*air*_, of integrated Arctic sea ice volume (*V*_*SI*_) in September 2006 to surface air temperature (*T*_*air*_) at a lead time of (a) 0, (b) 4, (c) 9, and (d) 12 months. Positive values (red) indicate where warming drives an increase in September 2006 ice volume. The solid black line delineates the ice edge at the given lead times. Sensitivities are plotted per unit area and shown only for latitudes north of 60°N. The sensitivity shown in panel (d) informs (e) the *T*_*air*_ perturbation applied in WARM.

Below, we show that our method is powerful in allowing us to unambiguously attribute ice growth to surface atmospheric warming—a counter‐intuitive scenario. The main limitation of our method arises from the entailed assumption that the ice response is linear, which can be validated with finite difference experiments. We find this assumption to be reliable for the response time and perturbation amplitude (Figure 1e) considered here (the reader is referred to Heimbach et al., 2010  for worked examples and in‐depth discussion).

## 3 Results

### 3.1 Timescales and Patterns of Sea Ice Volume Sensitivity

Sensitivity patterns of total Arctic sea ice volume averaged over September 2006 to *T*_*air*_ at four lead times are shown in Figure 1. Positive sensitivity indicates locations where atmospheric warming (at the indicated lead time) increases total sea ice volume in September 2006. At lead times of (a) 0, (b) 4, and (c) 9 months, the sensitivities are largely negative, consistent with the expectation that atmospheric warming drives sea ice melt. However, large regions of positive sensitivity are seen to *T*_*air*_ at 12 months lead time (i.e., the previous September, panel d), suggesting atmospheric warming in September may act to drive anomalous sea ice growth and relatively high sea ice volumes during September of the following year.

To understand this sensitivity, we perform a series of forward model perturbation experiments, informed by the sensitivity distributions shown in Figure 1. Since we are mostly interested in understanding how atmospheric warming drives sea ice growth at a later time, the perturbations we choose are patterns of *δT*_*air*_ > 0 that project exactly onto regions of positive sensitivity in September 2005 (Figure 1e). This also serves to show that the counter‐intuitive sensitivities are not merely artifacts of the adjoint model but exist in the forward model. The perturbation is imposed for 1 month, centered on 1 September 2005. We choose a maximum perturbation of 2°C. This choice is based on the maximum interannual variability inferred in this region from the JRA‐55 atmospheric reanalysis (Kobayashi et al., 2015). We emphasize that the pattern of the imposed perturbation is not informed by observations; our aim here is not to reconstruct observed variability in *V*_*SI*_ but to understand the counter‐intuitive sign of the sensitivity shown in Figure 1d. In the following we will refer to the unperturbed model integration as “CONTROL” and the integration with the imposed atmospheric warming perturbation as “WARM.”

### 3.2 Sea Ice Response to Local Atmospheric Warming

**Figure 2:**
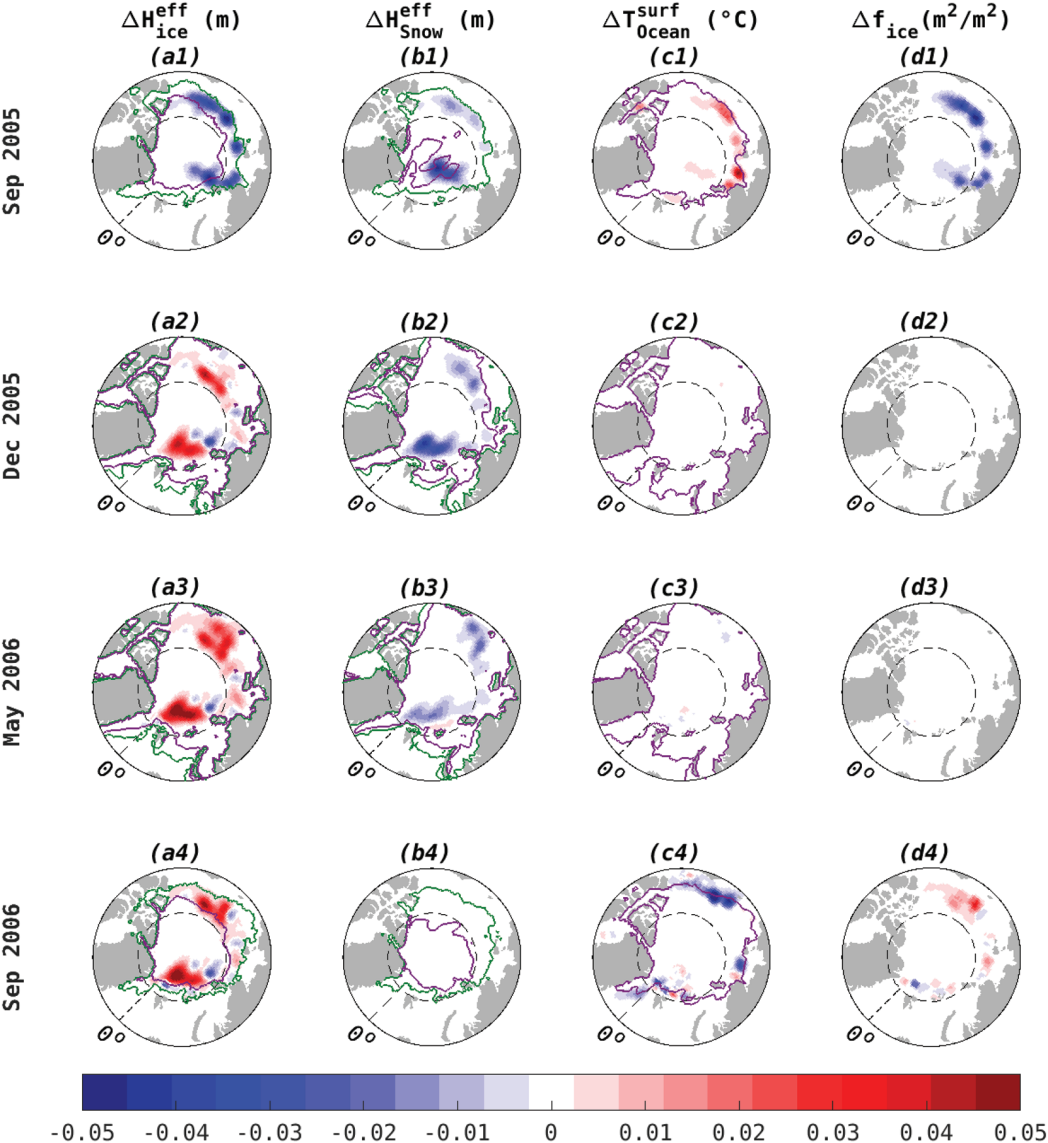
Difference between perturbed and unperturbed simulations (WARM‐CONTROL) for monthly averaged (a) effective ice thickness (m), with 80% (magenta) and 20% (green) ice cover contours, (b) effective snow height (m), with 1 mm (magenta) and 7 cm (green) snow thickness contours, (c) sea surface temperature (°C), with freezing temperature contoured, and (d) ice cover fraction. We show only the region north of 70°N, where differences are notable.

The impact of the atmospheric warming imposed in September 2005 on snow thickness, ice thickness, surface ocean temperature, and sea ice fraction is shown in Figure 2. The impact is assessed as the difference between the perturbed and reference experiments (WARM‐CONTROL) for different times following the perturbation. Immediately following the warming perturbation (i.e., within the first month), approximately 10 cm of ice and snow are melted locally (Figures 2a1 and 2b1), while the ocean surface warms from increased radiative and sensible heat flux (Figure 2c1), as expected.

Three months after the perturbation, in December 2005, the sea surface temperature is near freezing, and the ocean surface is fully covered by ice. At this time, CONTROL and WARM show identical ocean surface conditions (Figure 2c2), and there is less snow in WARM (Figure 2b2) due to prior melting. However, at this time, we observe increased ice volume in WARM (Figure 2a2), which survives the following melt season (Figure 2a3), persisting in September 2006 (Figure 2a4) and is accompanied by SST cooling (Figure 2c4).

**Figure 3:**
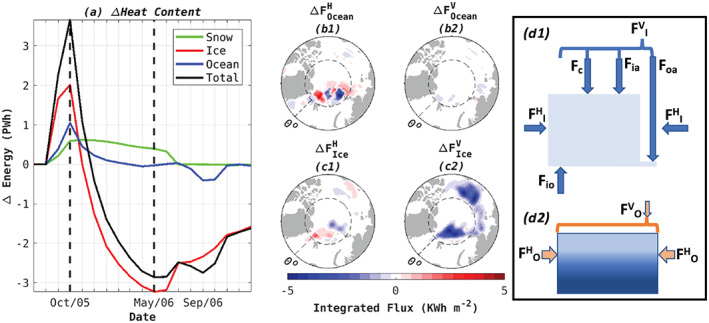
(a) Time evolution of the energy response (WARM‐CONTROL) for each component of the ice‐snow‐ocean system. (b, c) Difference (WARM‐CONTROL) in time‐integrated (b1, c1) horizontal and (b2, c2) vertical fluxes in (b1, b2) ocean and (c1, c2) ice reservoirs. The time integral is performed over the period delineated by the black dashed lines in panel (a). Fluxes contributing to the heat budget are sketched here for the (d1) ice and (d2) ocean reservoirs; terms are described in the main text. For each component in the system, the reservoir capacity (i.e., limits for the vertical integral in Figure 3a) corresponds to the full depth of the component.

To explore the fate of the additional heat added from the atmosphere, we examine the difference (WARM‐CONTROL) in the evolution of the heat budget for the ocean‐ice‐snow system. Time series of heat content change, integrated over the full depth of the relevant (snow, ice, or ocean) component is shown in Figure 3a. Following the warming perturbation, the heat content of the ice, snow, and ocean reservoirs increases as expected. Over the following few months, the heat reservoirs of the snow and ocean slowly return to their unperturbed (CONTROL) values. In contrast, the sea ice reservoir in WARM loses more heat from September 2005 onwards, compared to CONTROL, reaching a minimum relative heat content in May 2006, when maximum relative energy loss in terms of gains in ice volume occurs.

The pattern of the imposed warm perturbation, Figures 3b and 3c, is inferred from the difference (WARM‐CONTROL) in net horizontal and vertical ocean and ice heat fluxes integrated over the period of significant heat loss from the ice‐snow reservoir (1 October 2005–1 May 2006). Positive (negative) heat fluxes are defined as the vertically integrated convergence (divergence) of heat into each numerical cell. A schematic of the individual flux contributions is shown in Figures 3d1 and 3d2. Vertical heat fluxes in the ocean are almost identical for WARM and CONTROL (Figure 3b2). Due to changes in sea ice distribution, there are regions, for example, north of Fram Strait, with non‐negligible differences in the horizontal ocean heat fluxes (Figure 3b1). In the same region, there is also a perturbation of the horizontal energy flux in the ice component (Figure 3c1), associated with increased wind‐driven export of ice through Fram Strait (not shown). The key result is the large decrease in vertical heat flux downward into the ice in WARM relative to CONTROL (Figure 3c2). The change in heat content of the total ice‐snow‐ocean system is dominated by changes in the ice reservoir (Figure 3a), associated with reduced vertical heat fluxes in the sea ice component (Figure 3c2).

**Figure 4:**
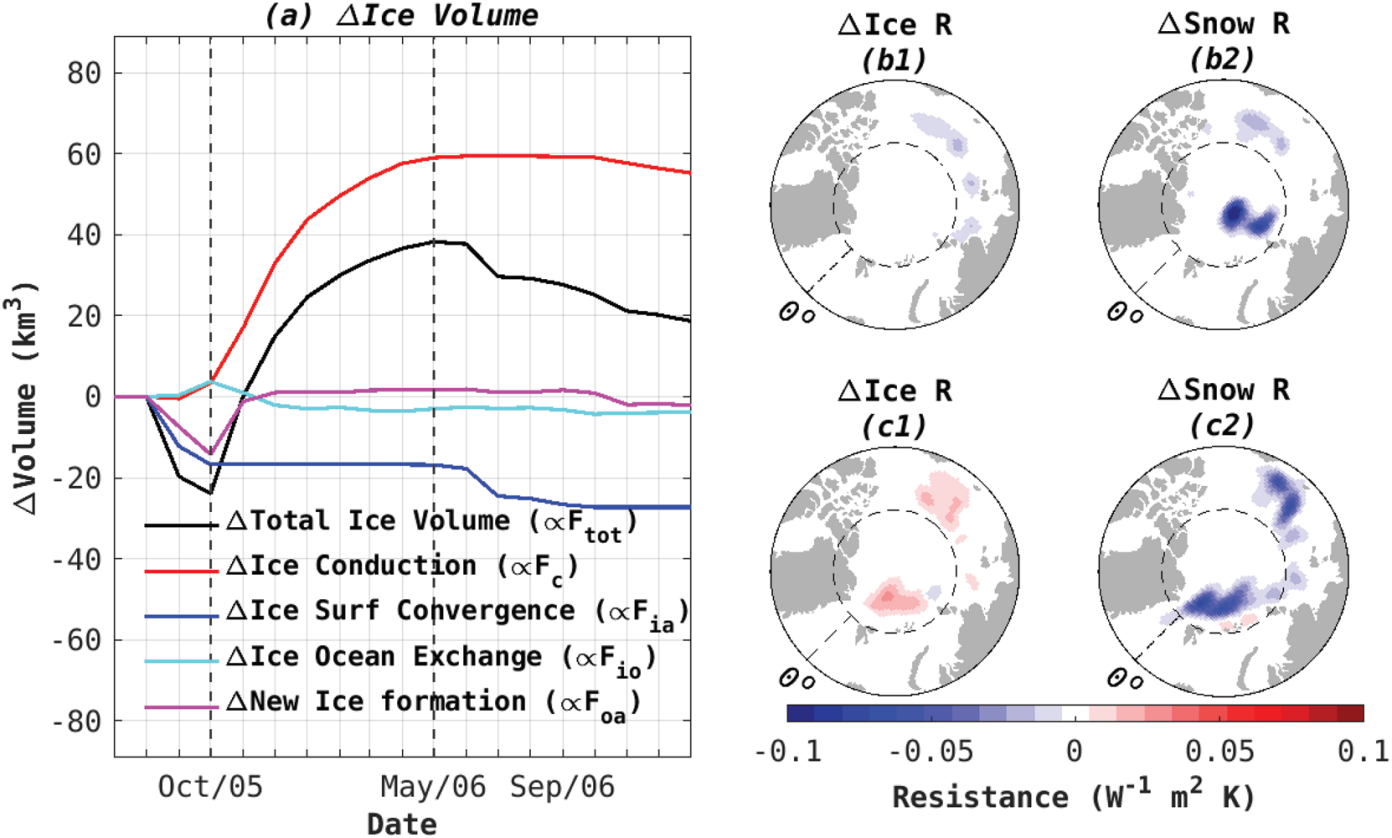
(a) Time evolution of ice volume response (WARM‐CONTROL) associated with each component of the perturbed sea ice vertical heat flux. (b, c) Difference (WARM‐CONTROL) in (b1, c1) ice resistance and (b2, c2) snow resistance on (b1, b2) 1 October 2005 and (c1, c2) 1 May 2006. These dates are shown by the black dashed lines in panel (a) and delineate the period of significant sea ice loss.

The net vertical heat flux (*F*_*tot*_) leading to changes in ice volume results from the following processes: (1) conduction (*F*_*c*_), (2) ice surface convergence (*F*_*ia*_), (3) ice‐ocean exchange (*F*_*io*_), and (4) ocean‐atmosphere exchange where new ice is forming (*F*_*oa*_) Figures 3d1 and 3d2). In this decomposition, *F*_*c*_ is balanced by sea ice production without changing the ocean mixed layer temperature. The magnitude of this flux is proportional to the effective conductivity of the ice‐snow system and also to the temperature gradient across the combined ice and snow layers (Hibler, 1979). *F*_*ia*_ depends on the temperature gradient across the ice‐atmosphere interface. In absence of flooding, sea ice can only grow from below, and *F*_*ia*_ only acts as a sink of ice volume, associated with melting of ice due to heat and radiation exchanges with the atmosphere. To better understand the enhanced vertical heat flux out of the ocean (Figure 3c2) in WARM, we investigate the change in sea‐ice volume associated with each of these four contributions to the vertical heat flux (Figure 4).

An enhanced conductive heat flux (*F*_*c*_) accounts for the increase in Arctic ice volume that occurs as a lagged response to atmospheric warming (Figure 4a). The anomalous ice volume loss corresponding to the perturbed atmosphere‐ice heat flux (*F*_*ia*_) opposes this conductive heat loss but is insufficient to offset it. Δ*F*_*ia*_ is unchanged throughout the ice growth season. It drives a small increase in melting at the start of the following melt season, associated with increased ice cover. In contrast, Δ*F*_*c*_ monotonically increases for the year following the warming perturbation, persisting through the following melt season.

To make the relation between growth of the snow/ice layers and increased buffering of conductive heat fluxes explicit, it is useful to frame our discussion below in terms of the “thermal resistance” in place of thermal conductivity. Snow and ice thermal conductivities are empirically derived constants, defined per unit length and related to their material properties. Conductivity values of 2.16 and 0.31 (W m^−1^ K^−1^) are widely used in climate models for ice and snow, respectively (Lecomte et al., 2013; Rae et al., 2014). Associated thermal resistance is defined as the reciprocal of the thermal conductivity multiplied by the layer thickness. The benefit of our resistance framework is best seen in analogy to a simple electrical circuit: Consider electrical resistors, which when placed in series carry the same current. Similarly, our ice and snow layers convey the same vertical heat flux sequentially. The total resistance of the electrical resistors in series is simply the sum of their individual resistances. Analogously, the snow and ice resistances in our system are additive, but their (reciprocal) conductivities are not. The resistance of snow per meter (3.22 W^−1^ m K) is approximately seven times larger than that of ice per meter (0.46 W^−1^ m K). Considering a case where 10 cm of snow is lost through surface melt as an example, it is now easy to see that 70 cm of ice would need to form via basal freezing in order to retain the same total insulating effect, highlighting the efficacy of snow as a thermal buffer.

A comparison of the difference (WARM‐CONTROL) in snow and ice resistance at the start (Figures 4b1 and 4b2) and end (Figures 4c1 and 4c2) of the freezing period reveals that snow melt is essential for driving the enhanced conductive heat flux (Figure 4a) and ice growth (Figures 2a2–2a4) in WARM. Immediately following the atmospheric temperature perturbation in September 2005, snow and ice both melt, reducing their total resistance. Reduced resistance allows faster conductive heat loss, accelerating basal ice growth. At the end of the freezing period (April 2006), despite thicker sea ice in WARM relative to CONTROL (Figure 4c1), the total resistance is reduced due to the much reduced resistance of a thinner snow layer (Figure 4c2). The fact that snow resistance greatly exceeds ice resistance is therefore critical in sustaining enhanced multi‐annual ice growth in response to atmospheric warming and initial melt of both snow and ice in the Arctic Basin.

To ascertain the key role of snow in sustained ice growth in response to atmospheric warming, we repeated the adjoint sensitivity calculations in another simulation where snow was removed from the initial conditions and precipitation was turned off at runtime to prevent new snow from forming. In this snow‐free Arctic experiment, the positive sensitivity of ice volume with respect to air temperature seen in WARM is absent (figure not shown). Atmospheric warming leads to enhanced sea ice melt at short lag. The sea ice slowly recovers through the following growth season, never overshooting its original (unperturbed) volume.

## 4 Discussion

Using a coupled ocean‐sea ice GCM and its adjoint, we have investigated the space and time‐evolving sensitivity of total sea ice volume in the Arctic to changes in near‐surface atmospheric temperature. Counter‐intuitively, we find that near‐surface atmospheric warming can lead to lagged sea ice growth. Specifically, we identify extended regions where warming at the end of the melt season enhances subsequent ice growth.

The responsible mechanism is a negative conductivity feedback, wherein enhanced summer melt preconditions the system for faster growth in the following winter via enhanced conductive heat loss. Previous studies have focused on the importance of early ice thinning in this feedback (i.e., the negative sea ice‐conductivity feedback, Bitz & Roe, 2004; Notz, 2009). Our novel contribution is to highlight the key role of snow melt early (around September) in the sea ice‐growth season, which drastically enhances conductive heat loss and is essential for growing sea ice later in the season (from November onwards, Figure 4a), beyond its volume prior to the onset of early season snow melt. Previous work has illustrated that the sea ice response to warming depends on the mean state (i.e., ice thickness and area) (Massonnet et al., 2018). Here we emphasize that snow thickness is the critical control on the mean state due to (1) the substantially larger thermal resistance of snow compared to ice and (2) the fact that snow melt will always precede underlying sea ice melt in response to atmospheric warming.

Unambiguous detection of negative snow‐melt‐conductivity feedback is enabled through calculation of time and space‐varying ice volume sensitivities to surface air temperature. To our knowledge, this is the first published adjoint‐based investigation suggesting that warming can lead to sea ice growth. We note this feedback is not evident in the adjoint‐based assessment of Arctic ice volume change by Koldunov et al. (2013), who apply notable time and/or space smoothing to their presented sensitivities to focus on drivers of low frequency trends over large regions. Snow thickness (and its spatial distribution) is not considered as a significant contributing factor in the 2007 sea ice area analysis by Kauker et al. (2009). Our findings are consistent with the adjoint‐based assessment of Arctic ice export by Heimbach et al. (2010), who attribute reduced export through Lancaster Sound in the Canadian Arctic to suppression of ice growth by snow accumulation in early winter.

We have focused on the response of Arctic sea ice volume to atmospheric warming. We did not address the response of Arctic ice volume to near‐surface ocean warming, although this has received significant attention in the literature in recent years, amid growing evidence of enhanced upper ocean radiative heating of the Arctic Ocean (Moore et al., 2016; Timmermans et al., 2018). For example, Petty et al. (2018) have reported sea ice growth as a lagged response to ocean and atmosphere warming in the Community Earth System Model Large Ensemble. In our experiments, SST warming is observed in some regions. However, the contribution to conductivity from the resulting basal melt is negligible. We conclude that the key physical mechanism underlying this correlation is between atmospheric warming and lagged sea ice growth, consistent with Stroeve et al. (2018). Interestingly, both Stroeve et al. (2018) and Petty et al. (2018) project a future weakening of their inferred correlation between conditions at the start of the freezing season and the sea ice growth rate from which they infer a slowdown of the negative conductive feedback.

To elucidate the importance of the insulating snow layer, we imposed an artificial surface warming perturbation (Figure 1e), purposefully constructed to activate ice growth, and traced its pathway through the system (Figures 3 and 4). In some areas, the perturbation resulted in an initial thinning by up to 7 cm (Figure 2a1) followed by enhanced growth of up to  14 cm (Figure 2a2). Positive thickness perturbations of up to 7 cm still persist in parts of the Arctic 1 year after the warming is applied (Figure 2a4). In future work we will examine the linearized response to realistic atmospheric forcing patterns to estimate the relative importance of the snow‐melt‐conductivity feedback in observed Arctic ice volume change. Another important avenue for future research is to explore the dependence of the snow‐conductivity feedback on the initial snow/ice state and to better understand the two hotspots of strong positive sensitivity in Figure 1d.

Snow thickness at the beginning of the sea ice growth season determines the strength of the subsequent conductivity feedback described in this study. In turn, snow thickness depends on a variety of factors (see Figure 1 Webster et al., 2018 of for an illustrated review). These include regional atmospheric circulation patterns delivering snow precipitation and driving snow melt (Hegyi & Taylor, 2016, 2017), the state of the existing sea ice, and dynamical mechanisms such as wind‐blown redistribution and sea ice flooding, with the latter becoming a more frequent occurrence in the Arctic (Provost et al., 2017) as the ice thins. The ability of current climate models to capture these dependencies and reliably simulate snow accumulation and melt cannot be assessed in the absence of detailed observations. Existing constraints have been provided by regional in situ (Warren et al., 1999) and airborne radar (Kurtz & Farrell, 2011) campaigns. Although these have improved our understanding of local ice‐snow evolution, together they reveal significant time variability and spatial heterogeneity in snow processes (Haas et al., 2017; Rsel et al., 2018). As a result, the latest IPCC Special Report on the Ocean and Cryosphere in a Changing Climate has placed low confidence in current and future snow trends on Arctic sea ice (Meredith et al., 2019). Our results highlight the need for improving large‐scale constraints of snow conditions on sea ice via remote sensing (Kwok & Markus, 2018) to better understand and skillfully model the rapidly changing Arctic environment.

## Supporting information

Supporting Information S1

Data Set S1

Data Set S2

Data Set S3

Data Set S4

## Data Availability

The ASTE data are obtained from the website (https://web.corral.tacc.utexas.edu/OceanProjects/ASTE/).
