# Supplementary material for: Atmospheric Warming Drives Growth in Arctic Sea Ice: A Key Role for Snow
Source: Geophys Res Lett. 2020 Oct 24;47(20):e2020GL090236. doi: 10.1029/2020GL090236 (PMC7685162; doi:10.1029/2020GL090236)
Supplement: Supplementary file 1 — Supporting Information S1 [file GRL-47-e2020GL090236-s001.docx]

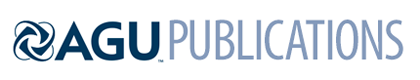


*Geophysical Research Letters*

Supporting Information for

**Atmospheric warming drives growth in Arctic sea-ice: a key role**

A. Bigdeli^1^, A. T. Nguyen^1^, H. R. Pillar^1^, V. Ocaña^1^, P. Heimbach^1,2,3^

^1^Oden Institute for Computational Engineering and Sciences, University of Texas at Austin

^2^Jackson School of Geosciences, University of Texas at Austin

^3^Institute for Geophysics, University of Texas at Austin

**Introduction**

The description of the supporting information is as follows:

- The Model outputs required to reproduce colormaps in figures 1 to 4, in ascii format.
- The data are formatted as latitude-longitude-variable text files.
- Each file consists of the headers and units (first line) followed by data.
